# Angiotensin II Causes Apoptosis of Adult Hippocampal Neural Stem Cells and Memory Impairment Through the Action on AMPK‐PGC1α Signaling in Heart Failure

**DOI:** 10.1002/sctm.16-0382

**Published:** 2017-02-28

**Authors:** Min‐Seok Kim, Geun‐Hee Lee, Yong‐Min Kim, Byoung‐Wook Lee, Hae Yun Nam, U‐Cheol Sim, Suk‐Jung Choo, Seong‐Woon Yu, Jae‐Joong Kim, Yunhee Kim Kwon, Seong Who Kim

**Affiliations:** ^1^ Department of Internal Medicine University of Ulsan College of Medicine Seoul Korea; ^2^ Department of Biochemistry and Molecular Biology, Asan Medical Center University of Ulsan College of Medicine Seoul Korea; ^3^ Department of Biology and Department of Life and Nanopharmaceutical Sciences Kyunghee University Seoul Korea; ^4^ Department of Thoracic and Cardiovascular Surgery, Asan Medical Center University of Ulsan College of Medicine Seoul Korea; ^5^ Department of Brain and Cognitive Sciences Daegu Gyeongbuk Institute of Science and Technology (DGIST) Daegu Korea

**Keywords:** Angiotensin, Cell biology, Cardiac, Neural stem cell

## Abstract

Data are limited on the mechanisms underlying memory impairment in heart failure (HF). We hypothesized that angiotensin II (Ang II) may determine the fate of adult hippocampal neural stem cells (HCNs), a cause of memory impairment in HF. HCNs with neurogenesis potential were isolated and cultured from adult rat hippocampi. Ang II decreased HCN proliferation in dose‐ and time‐dependent manners. Moreover, Ang II treatment (1 µM) for 48 hours induced apoptotic death, which was attenuated by pretreatment with Ang II receptor blockers (ARBs). Ang II increased mitochondrial reactive oxygen species (ROS) levels, which was related to mitochondrial morphological changes and functional impairment. Moreover, ROS activated the AMP‐activated protein kinase (AMPK) and consequent peroxisome proliferator‐activated receptor gamma coactivator 1‐alpha (PGC1α) expression, causing cell apoptosis. In the HF rat model induced by left anterior descending artery ligation, ARB ameliorated the spatial memory ability which decreased 10 weeks after ischemia. In addition, neuronal cell death, especially of newly born mature neurons, was observed in HF rat hippocampi. ARB decreased cell death and promoted the survival of newly born neural precursor cells and mature neurons. In conclusion, Ang II caused HCN apoptosis through mitochondrial ROS formation and subsequent AMPK‐PGC1α signaling. ARB improved learning and memory behaviors impaired by neuronal cell death in the HF animal model. These findings suggest that HCN is one treatment target for memory impairment in HF and that ARBs have additional benefits in HF combined with memory impairment. Stem Cells Translational Medicine *2017;6:1491–1503*

**Significance Statement**

This article investigated for the first time function and survival of hippocampal neural stem cells (HCNs) as a cause of memory impairment in heart failure (HF) in a carefully defined fashion. Impaired memory in HF animals was accompanied by hippocampal neuronal cell death, which was abrogated by angiotensin II (Ang II) receptor blocker. This article also demonstrated in mechanistic detail that a causative role of Ang II‐induced mitochondrial reactive oxygen species in regulating hippocampal neuronal cell death through its action on the AMPK‐PGC1α‐Bax signaling. Ang II receptor blocker and antioxidant markedly abolished the cell death and memory impairment in HF. Thus, this article revealed so far unappreciated link between HF‐associated memory impairment and Ang II‐induced cell death of HCNs, which may open up new therapeutic targets and tools for memory impairment in HF.

## Introduction

Cognitive impairment has been considered to be one of the most important comorbidities in heart failure (HF) because it leads to late recognition of HF symptoms and poor compliance to HF treatment. The functional deficit was associated with a decrease in the quality of life, increased risk of frailty, and poor survival in HF patients 1, 2. Cognitive impairment includes multiple domains such as memory, attention, executive functioning, psychomotor speed, language, and visuospatial ability and there is no denying the fact that memory functioning is one of the primary factors among various cognitive domains 3. However, the exact neuropathological cause of memory impairment accompanying HF has not yet been determined until now. Some MRI studies in HF patients demonstrated atrophy of the medial temporal lobe of the brain. This lobe includes the hippocampus, an important brain lesion for maintaining memory function 4. The loss of hippocampal volume could be associated with neuronal loss or diminution of newly formed neurons.

Conventionally, it is thought that the generation of neurons develops in the early fetal stages. However, the generation of new neurons during the adult period has also been reported in humans 5. In particular, adult hippocampal neural stem cells (HCNs) have been investigated as a functional mediator of neurogenesis for memory processing 6. Experimental animal studies have shown that adult neurogenesis can be augmented by behavioral stimulations in which newly developed neurons should impact hippocampus‐dependent memory function 7. In addition, the ablation of adult neurogenesis by pharmacology or irradiation impaired hippocampus‐dependent memory function 8. However, there have been few studies on HCNs' role in HF.

There are some emerging evidences that angiotensin II (Ang II) plays a role in causing memory impairment in various cardiovascular disorders. Activation of the renin–angiotensin system by a transgenic animal model impaired memory function via stimulation of the Ang II receptor 9. Besides, Ang II receptor blockers (ARBs) attenuated memory decline in both diseased animal models and hypertensive patients 10, 11. However, whether Ang II might affect the fate or function of HCNs in HF remains unknown.

In the present study, we investigated the effect of Ang II on the survival and function of HCNs, which would be a cause of memory impairment in HF. Furthermore, we also demonstrated that the adverse effect of Ang II on HCNs was mediated by the production of reactive oxygen species (ROS) as a result of impaired mitochondrial biogenesis. Finally, we discuss the effects of HCNs in a HF animal model and the beneficial roles of ARBs.

## Materials and Methods

Detailed materials and methods are available in the Supporting Information data.

### Isolation and Characterization of HCNs

HCN cells were primarily cultured from 11‐week‐old female Sprague‐Dawley rats (*n* = 6, 170–200 g, Orient Co., Ltd., a branch of the Charles River Laboratories in Korea, http://www.orient.co.kr/common/main.asp) and characterized by immunocytochemistry with nestin staining, as previously described 12. All experiments were performed on these six different HCN batches (at passage 5–10).

### Generation of a HF Animal Model

A total of 32 male Sprague‐Dawley rats (Orient Co., Ltd., a branch of the Charles River Laboratories in Korea, http://www.orient.co.kr/common/main.asp) were used, each weighing 250–300 g. The animals were divided into the control (*n* = 8), the sham operation group (*n* = 8), the HF group (*n* = 8), and the HF plus Losartan group (*n* = 8). Anesthesia was achieved with an intraperitoneal injection of 0.25 ml of a 2:1:2 (vol/vol/vol) mixture of Zoletil 50 (40 mg/kg; Virbac Laboratories, Carros, France, https://www.virbac.com/home‐en.html), Xylazine (10 mg/kg; Bayer, Leverkusen, Germany, https://www.bayer.com/), and 0.9% (wt/vol) physiological saline. We generated the HF animal model with the 11‐week‐old rats by left anterior descending artery ligation. Ketoprofen, 5 mg/kg subcutaneously daily and amoxicillin (150 mg/kg) subcutaneously twice daily were administered for 3 days postoperatively for pain and as a prophylaxis against infection. Animals were allowed access to water and food ad libitum in a temperature controlled room on a 12‐hours light/dark cycle. All surgeries were performed in an environment that was largely specific pathogen free. Animals were managed according to the “Revised Guide for the Care and Use of Laboratory Animal Resources” revised and published by the National Institutes of Health, 1996 (National Academy Press 1996:1e119) and the study was approved by the review board of the Asan Medical Center (IACUC No. 2012‐02‐195).

A Losartan tablet was dissolved in water (100 mg/l) and supplied instead of pure water (ad libitum) for 10 weeks. The rats were then examined by behavioral tests of about 1 week. 5‐bromo‐2‐deoxyuridine (BrdU, 50 mg/kg, Sigma‐Aldrich, St. Louis, MO, USA, https://www.sigmaaldrich.com/) was injected intraperitoneally three times before the rats were sacrificed for an immunohistochemical analysis to assess the number of proliferated neural precursor cells, whereas rats sacrificed 4 weeks after five BrdU injections were used to assess the survival of the newly born neural precursor cells.

### Morris Water Maze Task

The Morris water maze test (*n* = 8 for each group) was conducted in a circular stainless pool 160 cm in diameter and 60‐cm tall with a white‐painted inner surface. The pool was filled to a depth of 50 cm with water that was maintained at 23.0°C ± 1.0°C. An invisible platform (15‐cm white circle) was submerged 1.0 cm below the water surface and placed in the center of the northeast quadrant. Each rat was given one trial per day for 3 days to find the hidden platform (training trial). A trial was initiated by randomly placing the rat in one of the four quadrants facing the pool wall, all four quadrants were used once every day. For each trial, the rat was allowed to swim for a maximum of 60 seconds to find the platform and to rest for 1 minute on the platform upon success. Escape latency per group was defined as the average time of four quadrant tests on training days. On the last day of the training trial, rats were subjected to a probe trial in which the platform was removed from the pool, and rats were allowed to swim for 60 seconds to search for the removed probe position. All swimming times and trial lengths were recorded and monitored via a video camera. Tracking was accomplished by following the trajectory of the white rat, which was indicated by a black point against the white background. Captured video pictures were analyzed by a video‐tracking system (Ethovision water maze program, Noldus Information Technology, Wageningen, Netherland, http://www.noldus.com/). Analyzed information were the swimming time in the target quadrant (the swimming time spent to find the hidden platform in the target quadrant, where the hidden platform was positioned), and the number of virtual platform crossings frequency to find the removed platform.

### Statistical Analysis

In vitro data were reported as the mean ± SD. All behavioral and cell count data were expressed as the mean ± SEM. Comparisons between two groups were performed with unpaired Student's *t* tests. A Kruskal‐Wallis test or one way analysis of variance (ANOVA) followed by the least significant difference (LSD) multiple comparison test were performed when multiple comparisons were made and when appropriate (Graph Pad Prism, version 5.01). *p* values of < .05 was considered statistically significant.

## Results

### Ang II Induced HCN Apoptotic Death

**Figure 1:**
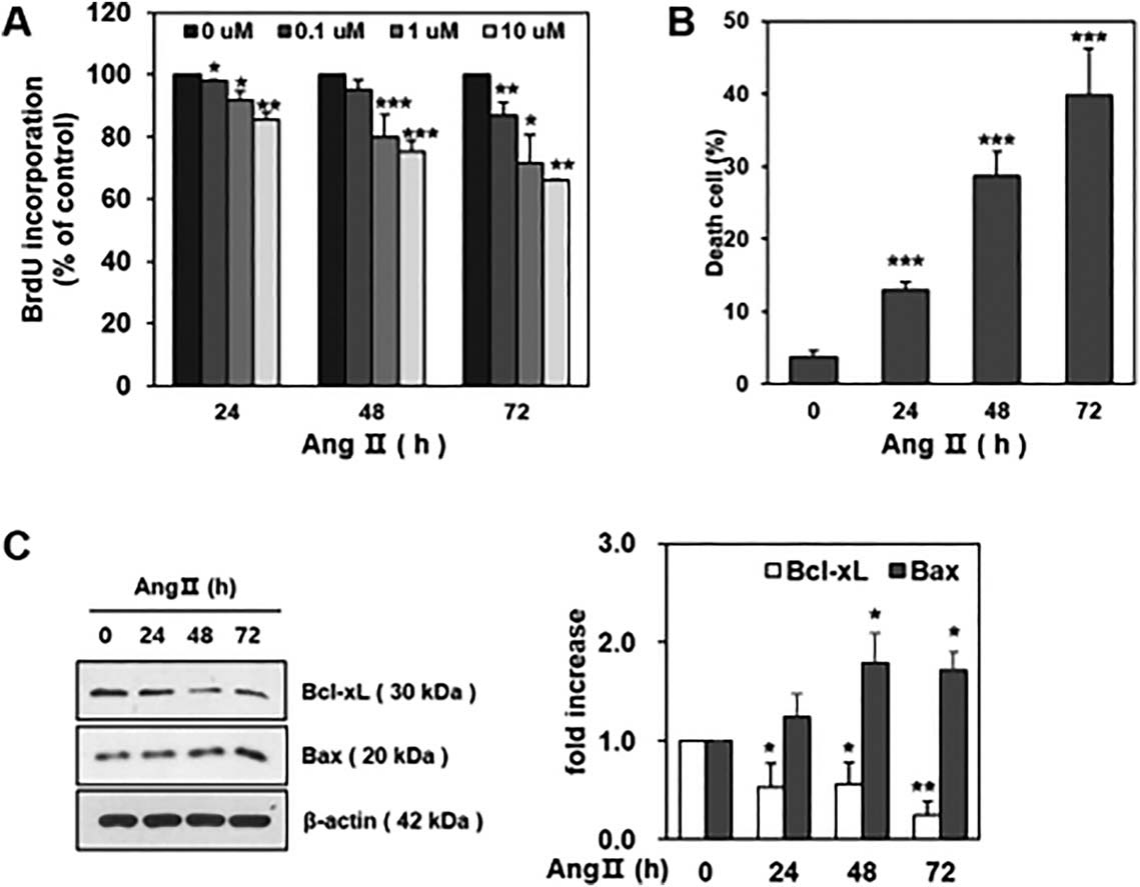
Ang II induced hippocampal neural stem cells (HCN) apoptotic death. **(A)**: Cell proliferation assay quantifies the incorporation of BrdU in HCNs. A significant decrease of BrdU incorporation was observed from 48 hours after Ang II treatment and at concentrations ≥1 µM (*, *p* < .05; **, *p* < .01; ***, *p* < .001, compared with the no treatment group). **(B)**: Trypan blue exclusion test for cell viability shows that Ang II treatment at 1 µM induced HCN cell death in a time‐dependent manner (***, *p* < .001, compared with the no treatment group). **(C)**: Representative immunoblot shows the protein expression of the pro‐apoptotic protein Bax and anti‐apoptotic Bcl‐xL after Ang II (1 µM) treatment in a time‐dependent manner (left figure). The blots were quantified using an image processing program (ImageJ) (right figure, *, *p* < .05; **, *p* < .01, compared with the no treatment group). *n* = 3/each experiment. Abbreviations: BrdU, 5‐bromo‐2‐deoxyuridine; Ang II, angiotensin II.

Quantitative real‐time PCR showed the constitutive expression of Ang II type‐1 (AT1R) and type‐2 receptors (AT2R) in cultivated HCNs (Supporting Information Fig. 1). To determine whether Ang II affected HCN proliferation, we used a BrdU cell proliferation assay. As shown in Figure. 1A, Ang II decreased HCN proliferation in a time‐dependent and dose‐dependent manner. A significant decrease was observed from 48 hours after Ang II treatment and at concentrations ≥1 µM. BrdU incorporations 48 hours after Ang II treatment were 95.2% ± 3.2% (*p=*.276), 79.9% ± 7.3% (*p* < .001), and 75.2% ± 3.6% (*p* < .001) of the control for 0.1 µM, 1 µM, and 10 µM, respectively. BrdU incorporations 72 hours after Ang II treatment were 87.0% ± 3.9% (*p* < .05), 71.4% ± 9.1% (*p* < .05), and 65.9% ± 0.3% (*p* < .01) of the control for 0.1 µM, 1 µM, and 10 µM, respectively.

Next, trypan blue exclusion tests were performed to estimate cell death. Ang II treatment at 1 µM induced HCN cell death (Fig. 1B). Dead cells accounted for 12.9% ± 1.1% of total cells (*p* < .001 compared with the no treatment group), 28.5% ± 3.5% (*p* < .001), and 40.0% ± 6.4% (*p* < .001) at 24, 48, and 72 hours after Ang II treatment, respectively. To confirm the mode of cell death in HCNs, we assessed the expression of pro‐ and anti‐apoptotic proteins using Western blotting. The expression of the pro‐apoptotic protein Bax increased and the anti‐apoptotic protein Bcl‐xL decreased in a time dependent manner after Ang II treatment (1 µM) (Fig. 1C).

### Ang II‐Induced HCN Apoptotic Death was Mediated by the Ang II Type‐1 Receptor

**Figure 2:**
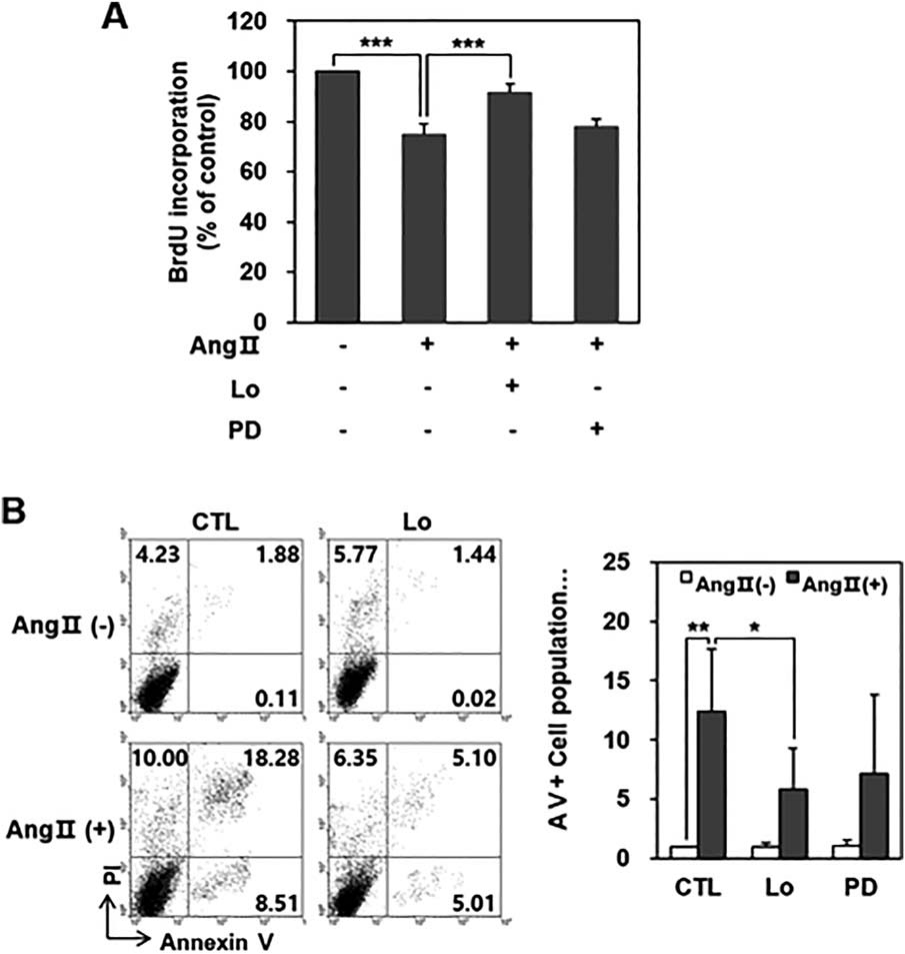
Ang II‐induced hippocampal neural stem cells (HCN) apoptotic death was mediated by Ang II type‐1 receptor. **(A)**: One hour pretreatment of Ang II type‐1 receptor blocker (Losartan, 1 µM) shows the recovery of BrdU decrease 48 hours after Ang II (1 µM) treatment. However, the Ang II type‐2 receptor blocker (PD123319) does not. BrdU incorporations were 91.4% ± 3.4% of the CTL in the Losartan pretreatment group (Lo, ***, *p* < .001, compared with the Ang II group) and 78.0% ± 3.0% in the PD123319 pretreatment group (PD, *p =* .225). **(B)**: A flow cytometry analysis shows that an increase in apoptosis induced by Ang II treatment (1 µM) for 48 hours was attenuated by Losartan, but not by PD123319. Representative flow cytometry image shows annexin V+ cells (26.79% of total cells) in the Ang II treatment group decreased in the Losartan pretreatment group (10.11% of total cells) (left figure). Ratios of the annexin V+ cell population (no treatment group was considered as 1) were 12.4 ± 5.3 (**, *p* < .01, compared with the no treatment group), 5.8 ± 3.5 (*, *p* < .05, compared with the Ang II treatment group), and 7.2 ± 6.6 (*p =* .224, compared with the Ang II treatment group) for the Ang II treatment group, Losartan pretreatment group, and PD123319 pretreatment group, respectively (right figure). *n* = 3/each experiment. Abbreviations: Ang II, angiotensin II; BrdU, 5‐bromo‐2‐deoxyuridine; CTL, control; Lo, Losartan pretreatment group; PD, PD123319 pretreatment group.

We tested a BrdU cell proliferation assay using pretreatment with selective ARBs, namely Losartan (Ang II type‐1 receptor‐selective) and PD123319 (Ang II type‐2 receptor‐selective) 1 hour before Ang II treatment. Ang II treatment (1 µM) for 48 hours decreased HCN proliferation and this was attenuated by Losartan pretreatment (1 µM) (Fig. 2A). However, the recovery of proliferative activity in HCNs was not observed by a PD123319 pretreatment (1 µM). A flow cytometry analysis showed that an increase in apoptosis induced by Ang II treatment for 48 hours was reduced by Losartan, but not with PD123319 pretreatment (Fig. 2B).

### Ang II Augmented the Production of Mitochondrial ROS Through the Ang II Type‐1 Receptor

**Figure 3:**
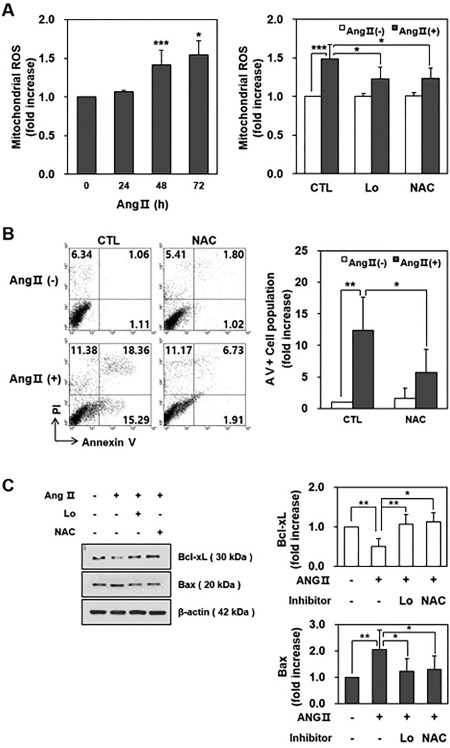
Ang II augmented the production of mitochondrial ROS through Ang II type‐1 receptor. **(A)**: MitoSOX fluorescence ratios were 1.07 ± 0.02 (*p* = .128, compared with the no treatment group), 1.41 ± 0.19 (***, *p* < .001), and 1.54 ± 0.18 (*, *p* < .05) at 24, 48, and 72 hours after Ang II treatment (1 µM), respectively (left figure). Increase in mitochondrial ROS induced by Ang II (1 µM) for 48 hours was attenuated by Losartan and NAC pretreatment. Mitochondrial ROS ratios were 1.49 ± 0.18 (***, *p* < .001, compared with the no treatment group), 1.23 ± 0.15 (*, *p* < .05, compared with the Ang II treatment group), and 1.24 ± 0.13 (*, *p* < .05, compared with the Ang II treatment group) for the Ang II treatment group, Losartan pretreatment group, and NAC pretreatment group, respectively (right figure). **(B)**: Representative flow cytometry image shows annexin V+ cells (33.65% of total cells) in the Ang II treatment group decreased in the NAC pretreatment group (8.64% of total cells) (left figure). Ratio of the annexin V+ cell population after Ang II treatment was 12.4 ± 5.3 (**, *p* < .01, compared with the no treatment group) and with a NAC pretreatment was 5.7 ± 3.7 (*, *p* < .05, compared with the Ang II treatment group) (right figure). **(C)**: Representative immunoblot shows that the protein expression of Bax increased and the expression of Bcl‐xL decreased from 48 hours after Ang II treatment (1 µM). These effects of Ang II were attenuated by Losartan or NAC pretreatment (left figure). The blots were quantified (right figure, *, *p* < .05 and **, *p* < .01). *n* = 3/each experiment. Abbreviations: Ang II, angiotensin II; CTL, control; Lo, Losartan; NAC, n‐acetyl cysteine; ROS, reactive oxygen species.

It has been known that Ang II increases the oxidative stress and mitochondrial dysfunction in other cells 13. Thus, we investigated whether Ang II might increase the expression of mitochondrial ROS in HCNs. MitoSOX was used as a specific fluorescent probe to measure mitochondrial ROS in the flow cytometry analysis of Ang II‐treated HCNs. Ang II (1 µM) increased MitoSOX fluorescence in HCNs in a time‐dependent manner (Fig. 3A). An increase in mitochondrial ROS induced by Ang II for 48 hours was attenuated by Losartan and n‐acetyl cysteine (NAC) pretreatment. When we measured the total intracellular ROS, the Ang II treatment showed a trend toward increased intracellular ROS in HCNs in a time‐dependent manner (Supporting Information Fig. 2). An increase in intracellular ROS induced by Ang II for 48 hours was reduced by Losartan and NAC pretreatment. In addition, based on the previous reports 14, 15, we tested whether Ang II treatment might induce activation of NADPH oxidase, through which total ROS subsequently increased in HCNs. The NADPH oxidase inhibitor (Apocynin) did not decrease HCN apoptotic death after Ang II treatment (Supporting Information Fig. 3).

To investigate whether Ang II‐induced mitochondrial ROS might contribute to apoptotic death, we additionally performed a flow cytometry analysis using annexin V in conjunction with propidium iodide (PI). In comparison with Ang II‐untreated HCNs, Ang II‐treated HCNs for 48 hours showed an increase in apoptosis, which was reduced by a NAC pretreatment (Fig. 3B). We also checked the amount of anti‐apoptotic protein Bcl‐xL and pro‐apoptotic protein Bax using Western blotting. The protein expression of Bax increased and the expression of Bcl‐xL decreased from 48 hours after Ang II treatment (Fig. 3C). These effects of Ang II were attenuated by Losartan or NAC pretreatment.

### Ang II Induced Structural and Functional Disruption of Mitochondria in HCNs

**Figure 4:**
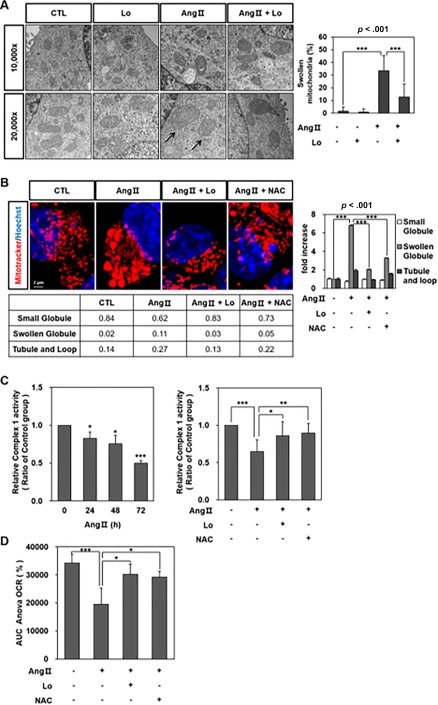
Ang II induced structural and functional disruption of mitochondria in hippocampal neural stem cells (HCNs). **(A)**: Electron microscopy shows that Ang II‐treated HCNs for 48 hours demonstrated swollen and disrupted mitochondria with a loss of outer and inner membrane structures and disordered cristae compared with the control or Losartan‐pretreated HCNs (left figure). The arrows indicate the structural disruption of mitochondria in HCNs. More than one hundred mitochondria per group were blindly analyzed by three independent examiners and the number of swollen mitochondria was determined. A swollen structure was barely found in the control or only Losartan‐treated HCNs when quantified (right figure). It was observed in 33.4% ± 12.1% of total mitochondria with Ang II‐treated HCNs and in 12.7% ± 10.2% with Losartan‐pretreated HCNs. ***, *p* < .001 by post hoc analysis using the least significant difference (LSD) test. **(B)**: Confocal laser scanning microscopy presents swollen mitochondria after Ang II treatment and this was reversed by Losartan or NAC pretreatment (left figure). In addition, quantitative analysis of mitochondrial morphology was performed (right figure). Fifty cells per group were blindly analyzed. The relative fold of each shape of mitochondria was shown as compared to the no treatment group. ***, *p* < .001 by post hoc analysis using the LSD test. (C) The activity of complex I decreased with Ang II administration in a time‐dependent manner, thereby confirming that Ang II can lead to mitochondrial dysfunction. Ratios of complex I activity compared with the no treatment group were 0.90 ± 0.01 (*, *p* < .05, compared with the no treatment group), 0.70 ± 0.16 (*, *p* < .05), and 0.44% ± 0.08% (***, *p* < .001) at 24, 48, and 72 hours after Ang II treatment, respectively. Losartan or NAC pretreatment restored the mitochondrial complex I activity level to near the control level. The ratio of complex I activity 48 hours after Ang II treatment (1 µM) was 0.65 ± 0.15 of the control (***, *p* < .001, compared with the no treatment group) and those with Losartan or NAC pretreatment were 0.86 ± 0.19 (*, *p* < .05, compared with the Ang II treatment group) and 0.90 ± 0.12 of the control (**, *p* < .01, compared with the Ang II treatment group), respectively. **(D)**: Total OCR (reserve capacity) was significantly lower in the Ang II treatment group and Losartan or NAC pretreatment recovered it. The mitochondrial reserve capacity was determined by calculating the total AUC of the FCCP‐stimulated respiration trace. The AUC ANOVA OCRs were 34.262% ± 3.099%, 19.524% ± 5.791% (***, *p* < .001, compared with the no treatment group), 30.293% ± 3.512% (*, *p* < .05, compared with the Ang II treatment group), and 29.189% ± 2.122% (*, *p* < .05, compared with the Ang II treatment group) for the control, Ang II treatment group, Losartan pretreatment group, and NAC pretreatment group, respectively. *n* = 3/each experiment. Abbreviations: Ang II, angiotensin II; AUC, area under the curve; CTL, control; Lo, Losartan; NAC, n‐acetyl cysteine; OCR, oxygen consumption rate.

The electron microscopic analysis identified that Ang II‐treated HCNs for 48 hours resulted in a swelling and disruption of the mitochondria with a loss of the outer and inner membrane structures and disordered cristae (Fig. 4A). These structural changes in mitochondria were also confirmed using MitoTracker Probes. A confocal laser scanning microscopic analysis showed the swollen mitochondria after Ang II treatment that was reversed by Losartan or NAC pretreatment (Fig. 4B). When we quantified the mitochondrial morphology, the number of swollen mitochondria increased after Ang II treatment, and reduced in Losartan or NAC pretreatment group. To further assess mitochondrial function, we measured the activities of the mitochondrial respiratory complex I. Complex I activity decreased with Ang II administration (Fig. 4C) in a time‐dependent manner, thereby confirming that Ang II can lead to mitochondrial dysfunction. Losartan or NAC pretreatment restored the mitochondrial complex I activity level to near the control level. Moreover, we measured the oxygen consumption rate (OCR) to investigate cellular bioenergetic responses to Ang II. Initially, a stable OCR baseline was established for 60 minutes, at which point Ang II (1 µM) was injected directly onto HCNs in the XF24 analyzer chamber. Changes in the OCR were monitored for a further 2 hours. Ang II decreased the total OCR in HCNs and the effect was attenuated by Losartan pretreatment (Fig. 4D).

### ROS Produced by Ang II Activated AMP‐Activated Protein Kinase Signaling and Induced Subsequent Overexpression of Peroxisome Proliferator‐Activated Receptor γ Coactivator 1α

**Figure 5:**
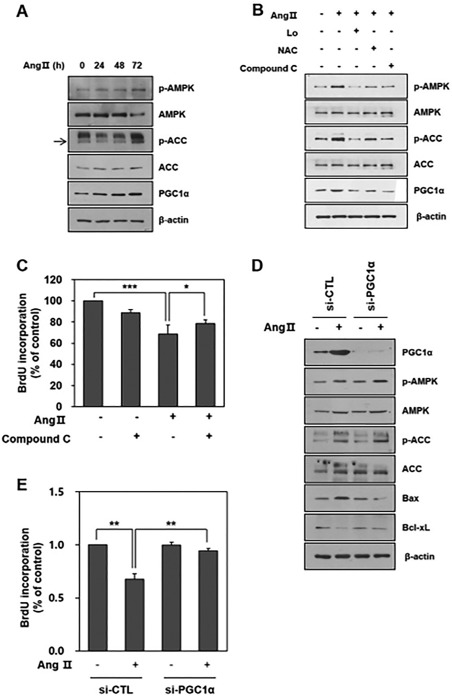
Ang II activated ROS‐the AMPK‐PGC1α sequentially. **(A)**: Representative immunoblot shows that Ang II treatment increased the expression of phosphorylated forms of AMPK and its substrate, ACC and PGC1α in a time‐dependent manner. **(B)**: Representative immunoblot shows that phosphorylated forms of AMPK and ACC decreased by Losartan, NAC, or Compound C pretreatment. The expression of PGC1α shows similar trends. **(C)**: A 1 hour pretreatment of Compound C shows an opposite trend toward a decreased proliferation of HCNs induced by Ang II treatment. BrdU incorporations were 71.1% of the control in the Ang II treatment group (***, *p* < .001, compared with the no treatment group) and 78.2% in the Compound C pretreatment group (*, *p* < .05, compared with the Ang II treatment group). **(D)**: Validated siRNA directed to PGC1α mRNA was used to knock down the PGC1α expression in HCNs. Successful knockdown of PGC1α was confirmed by the reduced protein levels. The expressions of the phosphorylated forms of AMPK and ACC 48 hours after Ang II treatment were not different between mock‐transfected (si‐CTL) and PGC1α siRNA‐transfected (si‐PGC1α) HCNs. However, the expression of Bax decreased and the expression of Bcl‐xL increased after Ang II treatment in si‐PGC1α cells compared to si‐CTL cells. **(E)**: BrdU incorporations decreased in si‐CTL cells (67.5% ± 5.3% of the control, **, *p* < .01, compared with the no Ang II treatment group) but not in si‐PGC1α cells (94.3% ± 2.1% of the control, **, *p* < .01, compared with Ang II treated si‐CTL cells). *n* = 3/each experiment. Abbreviations: ACC, acetyl‐CoA carboxylase; AMPK, AMP‐activated protein kinase; Ang II, angiotensin II; BrdU, 5‐bromo‐2‐deoxyuridine; Lo, Losartan; NAC, N‐acetyl cysteine; PGC1α, peroxisome proliferator‐activated receptor γ coactivator 1α.

The increase in mitochondrial ROS and decrease of the OCR raised our curiosity about the relationship between Ang II and AMP‐activated protein kinase (AMPK) signaling. AMPK is known as a sensor of energy balance activated by increases in AMP or by oxidant stress such as ROS 16. Thus, we investigated whether Ang II might activate the expression of AMPK in HCNs. Ang II increased the phosphorylation of AMPK and its substrate, acetyl‐CoA carboxylase (ACC), in a time‐dependent manner with Ang II treatment (Fig. 5A), which were attenuated by Losartan or NAC pretreatment (Fig. 5B). The AMPK inhibitor, Compound C, showed a trend toward a reverse of the decreased proliferation of HCNs induced by the Ang II treatment (Fig. 5C).

**Figure 6:**
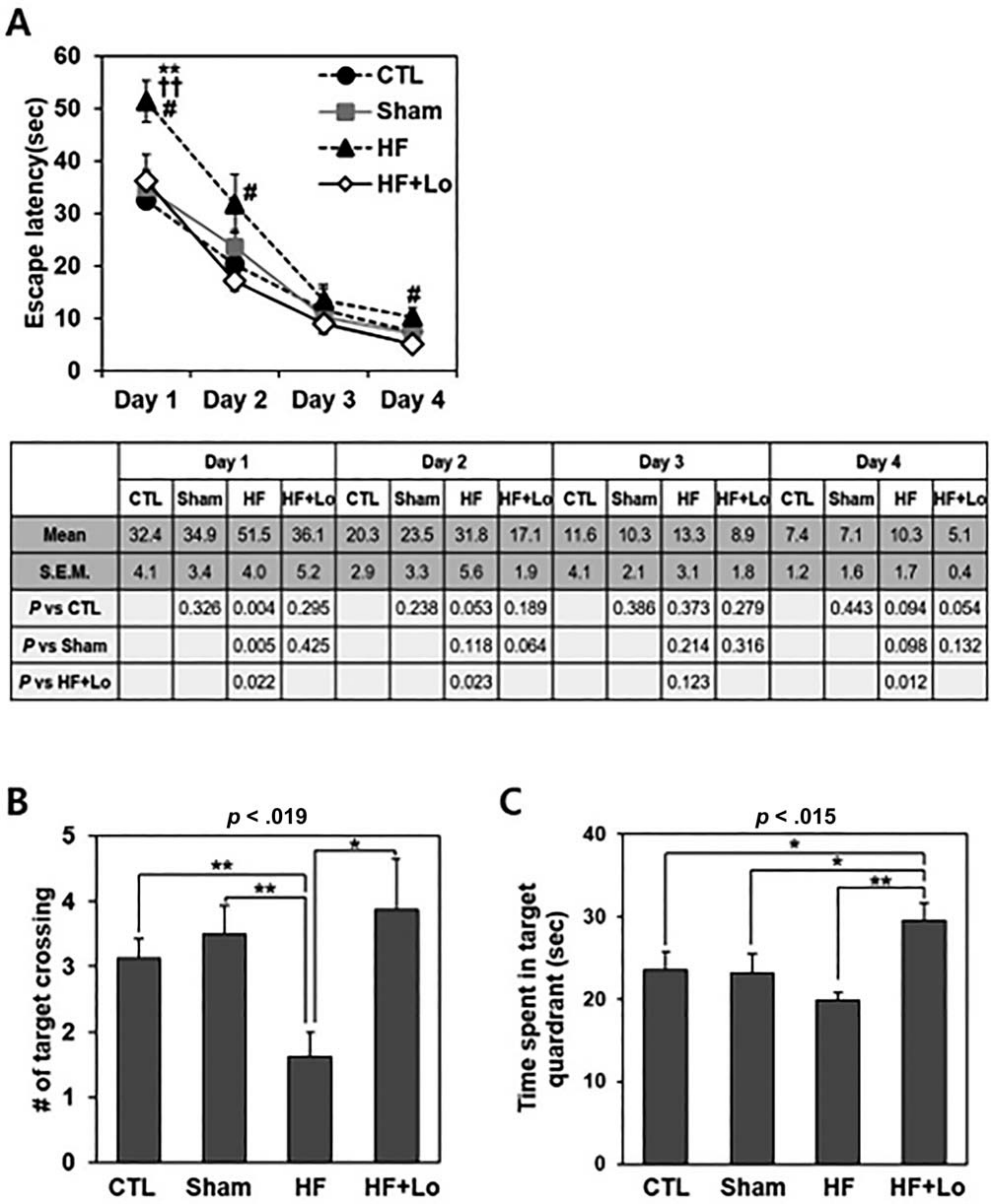
Learning and memory ability improved based on a Morris water maze test 10 weeks after the administration of Losartan in a rat model of HF. **(A)**: Mean escape latency time to find the hidden platform during four consecutive days of training trials. The escape latency time in the HF group was longer at the beginning than the control, sham, and HF+Lo groups (*p* = .020 by ANOVA) and the time of the HF+Lo group showed a decreased tendency compared with that of the HF group from the second and fourth days. **, *p* < .01, compared with the control group, ^††^, *p* < .01, compared with the sham group, and ^#^, *p* < .05, compared with the HF+Lo group by post hoc analysis using the least significant difference (LSD) test. **(B)**: Number of virtual platform crossings was measured and that was low in the HF group (*p* = .019 by ANOVA) and they recovered significantly in the HF+Lo group more than in the control and sham groups. **(C)**: Swimming time in the target quadrant in 60 seconds probe trials with no platform was also low in the HF group (*p* = .015 by ANOVA) and they recovered in the HF+Lo group more than in the control and sham groups. Data represent the mean ± SEM *, *p* < .05; **, *p* < .01 by post hoc analysis using the LSD test, *n* = 8 per group. Abbreviations: CTL, control; HF, heart failure; Lo, Losartan.

Meanwhile, we also investigated the expression of peroxisome proliferator‐activated receptor γ coactivator 1α (PGC1α), a key regulator of mitochondrial biogenesis and function in AMPK downstream signaling 17. Moreover, a recent study demonstrated that it showed a pro‐apoptotic effect mediated with Bax 18. Therefore, we investigated whether the effect of Ang II on mitochondrial dysfunction in HCNs might be mediated by PGC1α and Bax. The protein expression of PGC1α increased in a time‐dependent manner with Ang II treatment (Fig. 5A) and the expression was decreased by Losartan or NAC pretreatment (Fig. 5B). We also compared the apoptotic effect of Ang II with pioglitazone which is known to increase the expression of PGC1α 19. Pioglitazone decreased HCN proliferation in a dose‐dependent manner (Supporting Information Fig. 4A). Ang II treatment also decreased HCN proliferation. The expression of PGC1α and Bax increased after Ang II and pioglitazone treatment. Ang II and pioglitazone showed an additive effect in increasing PGC1α expression (Supporting Information Fig. 4B). However, pioglitazone did not show additive effects on HCN proliferation with Ang II in the BrdU analysis.

In addition, we blocked the signal of PGC1α using siRNA. Validated siRNA directed to PGC1α mRNA was used to knock down the PGC1α expression in HCNs (Fig. 5D). A successful knockdown of PGC1α was confirmed by the decreased protein levels. The expressions of the phosphorylated forms of AMPK and ACC 48 hours after Ang II treatment were not different between mock‐transfected (si‐CTL) and PGC1α siRNA‐transfected (si‐PGC1α) HCNs. However, the expression of Bax decreased and the expression of Bcl‐xL increased after Ang II treatment in si‐PGC1α cells compared to si‐CTL cells. These suggest that PGC1α is a downstream effector of AMPK for Ang II induced apoptosis. Besides, BrdU incorporations decreased in si‐CTL cells but not in si‐PGC1α cells (Fig. 5E).

### Spatial Memory Impaired in a HF Animal Model and ARB Ameliorated the Memory Behavior

To determine whether HF causes memory impairment and neuronal loss, we first examined spatial memory tested by the Morris water maze in a HF animal model which was generated by left anterior descending artery ligation (HF group). The same surgery protocol without artery ligation was carried out for sham group. Echocardiography performed 4 weeks after HF generation showed that left ventricular end‐systolic dimension (LVESD) and LVESD increased in the HF group compared with the control or sham groups (LVESD, 35.1 ± 7.6 vs. 29.9 ± 9.3 vs. 63.9 ± 11.0 mm, *p* < .001 for control, sham, and HF groups, respectively: LVEDD, 63.9 ± 5.5 vs. 62.7 ± 5.7 vs. 82.0 ± 8.6 mm, *p* < .001). Moreover, LV ejection fraction (EF) decreased in the HF group showing the generation of LV remodeling and HF (LVEF, 81.0 ±7.6 vs. 85.9 ± 8.0 vs. 49.8% ± 10.6%, *p* < .001 for the control, sham, and HF groups, respectively). At 6 weeks after HF model generation, serum Ang II levels seemed to be higher in the HF group than in the control or sham groups, although it did not reach statistical significance (37.8 ± 7.2 vs. 47.5 ± 24.4 vs. 56.3 ±19.9 pg/ml, *p =* .147 for the control, sham, and HF groups, respectively). However, at 8 weeks after HF model generation, serum Ang II levels were higher in the HF group than in the control or sham groups (36.9 ± 14.6 vs. 34.4 ± 17.6 vs. 77.3 ± 55.5 pg/ml, *p* < .001 for the control, sham, and HF groups, respectively). This elevation of Ang II levels continued until 10 weeks after HF generation. Losartan was supplied instead of water (ad libitum) to HF rats for 10 weeks from 4 weeks after ischemia was induced (HF+Lo group) and then learning and memory ability were examined by conducting the behavioral tests.

In the training trial session of the Morris water maze test, the escape latency time for finding the hidden platform in the HF group took longer at the beginning than the control, sham, and HF+Lo groups (*p* = .020 by ANOVA). The escape latency time declined progressively during the training period of four consecutive days, but the time of the HF+Lo group showed a decreased tendency from the second to fourth days (Fig. 6A). In the probe trial session, the number of crossings across the target probe (*p* = .019) and swimming times within the target quadrant (*p* = .015) was low in the HF group and they recovered significantly in the HF+Lo group more than in the control and sham groups (Fig, 6B, 6C). We also examined the Y‐maze task to elucidate short‐term memory. The spontaneous alterations in 10 and 12 minute sessions were low in the HF group and showed an increased tendency in the HF+Lo group, although the results were not statistically significant compared with the control and sham groups (Supporting Information Fig. 5). These results indicate that HF causes memory loss in the animal model and that Losartan administration improves the learning and memory ability in behavioral tests.

### Impaired Memory in HF Animals Was Accompanied by Neuronal Cell Death and Losartan Ameliorated the Neuronal Cell Survival

**Figure 7:**
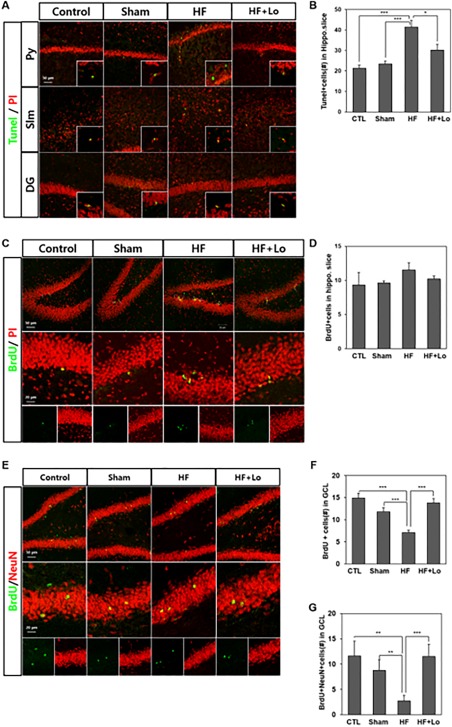
Losartan ameliorated the neuronal cell survival in memory impaired HF rats. **(A)**: TUNEL positive cells found in the entire hippocampal areas, including the Py, slm, and DG. Inlets show TUNEL positive cells with a higher magnification. **(B)**: Quantification of TUNEL‐positive cells. About twice the cell death in control group was found in the HF group but it was largely decreased in the Losartan treated group in the entire hippocampal area. **(C)**: BrdU‐labeled cells in the subgranular zone (SGZ) and granular cell layer (GCL) in the dentate gyrus 1 day after BrdU injections. **(D)**: Quantification of proliferating cell number. BrdU‐labeled cells, counterstained with PI were counted. Slightly more cells are labeled in the HF group but this is not significant. **(E)**: Survival of BrdU‐labeled cells in SGZ and GCL in the dentate gyrus 4 weeks after BrdU injections. BrdU positive cells doubly stained with NeuN are newly born neurons. They migrated into the GCL 4 weeks later. **(F)**: Quantification of surviving BrdU positive cell numbers 4 weeks after BrdU injections. **(G)**: Quantification of surviving BrdU positive neuronal cell numbers 4 weeks after BrdU injections. The survival of BrdU and/or NeuN positive cells was strikingly decreased in the HF group. Data are presented as the mean ± SEM. *, *p* < .05; **, *p* < .01; ***, *p* < .001, *n* = 4–8 per group. Abbreviations: BrdU, 5‐bromo‐2‐deoxyuridine; CTL, control; DG, dentate gyrus; HF, heart failure; Lo, Losartan; PI, propidium iodide; Py, pyramidal layer, slm, stratum lacunosum moleculare.

Next, we investigated whether memory impairment in HF animals is related to neuronal cell death in the hippocampal area by labeling brain slices with a TUNEL assay and BrdU (Fig. 7). When we immunostained brain slices with terminal transferase, and the whole area of the hippocampus was laser scanned under a confocal microscope, cell death was found in the pyramidal layer, stratum lacunosum molecular (slm) area, and dentate gyrus (Fig. 7A). Cell counts in the entire hippocampal area were similar to the control and sham groups but were almost twice as high in the HF group. In the HF+Lo group, the cell counts were about half of the HF group (Fig. 7B, ***, *p* < .001 compared with the HF group, *, *p* < .05 compared with the HF+Lo group). These results indicate that substantially more neuronal cells died in the hippocampus of the HF group, which recovered in the Losartan treated group.

To evaluate the survival rate of neuronal cells in the HF group, we injected BrdU (50 mg/kg) multiple times intraperitoneally and then sacrificed rats either 1 day later or 4 weeks later for an immunohistochemical analysis. At 4 weeks, new born cells labeled with BrdU in the subgranular zone (SGZ) of the hippocampus migrated into the granular cell layer (GCL) and some of them survived to differentiate into neurons. Therefore, we compared the numbers of BrdU positive‐newly born cells in the SGZ of 1‐day rats (Fig. 7C, 7D) with BrdU positive cells in 4 weeks rats to examine the survival rate of BrdU positive cells. Especially, double positive cells of BrdU and NeuN in the GCL of the HF group which had survived for 4 weeks after BrdU injections (Fig. 7E, 7F) were also compared with the control and HF+Lo groups. BrdU positive‐newly born neural precursor cells of the hippocampus in the HF group showed an increasing tendency in 1‐day rats, whereas 4 weeks later, there were about half the number of BrdU positive cells in the HF group compared with the control and sham groups, indicating that the survival rate of new born cells in the HF group was very low. In the HF+Lo group, BrdU‐labeled cell counts were almost similar to those of the control and sham groups. The survival rate of mature neuron cells (BrdU+NeuN‐double‐labeled cells) that had differentiated from the BrdU positive cells 4 weeks later were significantly lower (<25%) than that of new born cells (BrdU+cells). However, the survival of BrdU+NeuN‐labeled cells were almost the same as the control in the HF+Lo group (Fig. 7G). These results suggest that in the HF group, proliferation of new born HCNs were not lower than in the control group, but that those new born cells mostly die and the survival of new born neurons was substantially low, probably caused by Ang II receptor mediated cell death. Thus, the ARB is able to improve the survival of new born neurons and memory behavior in HF animal models.

## Discussion

In the present study, we investigated the underlying mechanism of memory impairment in HF and focused on the fate of HCNs under the stimulation of HF progression signals. For the first time, we found that HCNs expressed Ang II receptors, which makes Ang II stimulation reasonable. We demonstrated that Ang II induced apoptotic death of cultivated HCNs. This adverse effect of Ang II resulted from mitochondrial ROS production which activated AMPK‐PGC1α signaling and subsequent apoptotic pathways. In an animal model, HF caused memory loss and Losartan improved learning and memory behaviors impaired by neuronal cell death.

Ang II has been thought to be central in maintaining cardiovascular homeostasis and progressing cardiovascular disorders. Ang II's role in the brain has mainly focused on the influence on vascular resistance and body water and electrolyte balance. However, less attention has been paid to the substantial role of Ang II in memory processes. Recent evidences showed that continuous activation of the renin–angiotensin system in the brain contributed to the progression of memory impairment in animal models 9, 20. Moreover, some of ARBs or angiotensin converting enzyme inhibitors improved working memory irrespective of lowering blood pressure 10, 11. It was assumed that Ang II was involved in the production of important neurocytokines to maintain memory function such as acetylcholine, vascular endothelial growth factor, and n‐methyl‐d‐aspartate‐glutamate receptor 21, 22. However, the precise mechanism of why Ang II impaired memory function in cardiovascular disorders remains unclear. Our study for the first time showed that Ang II affects the fate and function of adult HCNs, causing memory impairment in HF.

In addition, we demonstrated that the effect of Ang II on HCN survival was mediated by AT1R, but not AT2R. A recent study showed AT2R's role on neuronal stem cell proliferation, although the stem cells were derived from the hippocampus of an embryo, not an adult 23. Thus, AT2R's role should be limited to embryonic development, but not adult neurogenesis. In addition, the study did not refute an argument that some selective AT1R blockers, namely ARB, improved memory dysfunction associated with cardiovascular disorders 10, 11. Our study proved that ARB recovered HCN survival in experimental and animal tests and memory impairment in behavioral tests of HF rats. Considering that the AT1R blocker relatively enhances the agonistic stimulation of AT2R, it is likely that Ang II‐induced HCN death is mainly mediated with AT1R.

It is well recognized that Ang II generates ROS, mainly mediated by activation of NADPH oxidase in the cardiovascular system 13. However, there are relatively few reports regarding Ang II's role on ROS production in the central nervous system. Furthermore, studies on the oxidative stress induced by Ang II in hypertension and HF mostly investigated neuronal activity and sympathetic regulation 24. We observed that Ang II augmented ROS generation in HCNs which mostly originated from mitochondria. ROS are generated in the brain from two major sources, NADPH oxidase and the mitochondrial electron transport chain 24. Our data showed that Ang II‐stimulated ROS was more dominantly produced from mitochondria than from NADPH oxidase in HCNs. The relative increase in ratio of ROS production after Ang II treatment was greater in mitochondrial ROS than in total ROS (Fig. 3, Supporting Information Fig. 2). Moreover, the NADPH oxidase inhibitor did not decrease HCN apoptotic death after Ang II treatment (Supporting Information Fig. 3). In addition, the increase of mitochondrial ROS production was associated with the impairment of mitochondrial morphology and function. What is the most important source for ROS in HCNs should be further investigated when they are stimulated with Ang II. However, the predominant involvement of mitochondrial ROS induced by Ang II in HCNs might be expected given the vulnerability of the brain to hypoxic insults and the importance of mitochondria in aerobic metabolism.

Another interesting finding herein was the observation that Ang II treatment increased the expression of AMPK‐PGC1α in HCNs with a concomitant increase in the proapoptotic protein Bax. We focused on these cellular signals from the morphological and functional changes in mitochondria. AMPK acts as a sensor of energy balance and handles mitochondrial biogenesis to produce ATP 16. However, previous studies demonstrated a conflicting issue that AMPK was activated for survival or apoptosis depending on various cell types and stimulations 25, 26. This opposite action of AMPK looks similar in PGC1α. PGC1α is a transcriptional coactivator of nuclear receptors responsible for the regulation of mitochondrial biogenesis and function 17. Thus, in general, its physiological role is known to increase the demand on mitochondria to produce ATP. However, the expression of PGC1α in the brain with high‐capacity mitochondrial systems has not been well described and its roles in adult neurogenesis also remain to be determined. A recent study showed that PGC1α prevented mitochondrial ROS production in the vasculature in response to Ang II 27. Conversely, another study demonstrated that PGC1α accumulated mitochondrial ROS mediated by proapoptotic Bax in cancer cells 18. Up to now, we do not know whether the expression of AMPK and PGC1α after Ang II treatment was increased because of their pro‐apoptotic role or compensatory mechanism in HCNs. However, our data showed that AMPK‐PGC1α activation by Ang II treatment was associated with decreased survival of HCNs. Moreover, Ang II treatment increased the AMPK‐PGC1α expression, which was reduced by Losartan or NAC pretreatment. Furthermore, Ang II treatment, like pioglitazone, activated PGC1α and Bax expressions. Blocking PGC1α with siRNA treatment attenuated Bax formation and Bcl‐xL reduction induced by Ang II. These findings suggest that AMPK‐PGC1α signaling plays an important role in controlling Ang II‐induced mitochondrial functional impairment, causing HCN apoptotic death.

This study has several experimental limitations. First, we did not measure cerebral blood flow in a HF rat model. Cerebral hypoperfusion is a feasible cause of memory impairment in parallel with a direct action of Ang II on HCN in HF 28. Thus, further investigation would be needed to verify a convincing link between cerebral blood flow and function of HCNs in HF. Second, we did not investigate whether systemic or local Ang II might affect memory functions differently. Many evidences suggested that the blood‐brain barrier in the hippocampus was interrupted and memory deficits developed during cardiovascular disorders by Ang II stimulation 29. Thus, the impact of systemic or local Ang II may not be significantly different. However, further research is warranted to focus on local Ang II's role and memory function. Last, in vivo neuropathological analysis mainly restricted to the HCNs might limit our conclusion because HF and cerebral perfusion deficit could cause a broad range of neuropathological changes beyond hippocampal area 30. To overcome this, an extensive analysis covering entire brain and additional experiments including specific knockout confirming the role of HCN in memory function need to be shown.

## Conclusion

Ang II caused HCN apoptosis through mitochondrial ROS formation and subsequent AMPK‐PGC1α signaling. These findings suggest that HCN is a treatment target for memory impairment in HF and that Ang II blockers with more brain penetration should be used for better outcomes.

## Author Contributions

M.‐S.K. and G.‐H.L.: conception and design, collection and/or assembly of data, data analysis and interpretation, manuscript writing; Y.‐M.K., B.‐W.L., U‐C.S., and Y.K.K.: provision of study material or patients, collection and/or assembly of data, data analysis and interpretation; H.‐Y.N. and S.‐W.Y.: data analysis and interpretation; S.‐J.C. and J.‐J.K.: administrative support, provision of study material or patients; S.W.K.: conception and design, final approval of manuscript.

## Disclosure of Potential Conflicts of Interest

The authors indicated no potential conflicts of interest.

## Supporting information

Supporting Information

Supporting Information

Supporting Information

Supporting Information

Supporting Information

Supporting Information
