# Supplementary material for: Angiotensin II Causes Apoptosis of Adult Hippocampal Neural Stem Cells and Memory Impairment Through the Action on AMPK‐PGC1α Signaling in Heart Failure
Source: Stem Cells Transl Med. 2017 Feb 28;6(6):1491–503. doi: 10.1002/sctm.16-0382 (PMC5689768; doi:10.1002/sctm.16-0382)
Supplement: Supplementary file 1 — Supporting Information [file SCT3-6-1491-s001.docx]

**Supplemental Materials and Methods**

**Isolation and characterization of HCNs**

HCN cells were primarily cultured from 11-week-old female Sprague-Dawley rats (n=6, 170–200g). Hippocampi were meticulously dissected bilaterally and fragmented into small pieces (<1 mm^3^). Tissues were digested for 30 min at 37°C in a Hanks balanced salt solution containing grade II neutral protease (1U/mL), papain (2.5U/mL), and Dnase I (250U/mL). They were disrupted by pipetting through a flame-polished pipette 2–3 times during digestion and rinsed once with phosphate-buffered saline (PBS, Amresco, Solon, OH, USA). After that, they were plated onto a 100-mm dish in Dulbecco’s modified Eagle’s medium (DMEM: Invitrogen, Carlsbad, CA, USA) supplemented with Ham’s F-12 medium (Invitrogen) and 10% bovine serum. All plates were coated with poly-L-ornithine (10 µg/mL for plastic plates and 50 µg/mL for glass; Sigma-Aldrich, St. Louis, MO, USA) and mouse laminin (5 µg/mL; BD Pharmingen, San Diego, CA, USA). The next day, the medium was replaced with DMEM with F-12 containing 1-mM L-glutamine (Invitrogen), 100-µg/mL streptomycin, 100-U/mL penicillin (Invitrogen), 20-ng/mL bFGF (Invitrogen), and our own-made N2 supplement, which includes 5-mg/l insulin (Sigma-Aldrich), 16-mg/l putrescine dihydrochloride (Sigma-Aldrich), 100-mg/l transferrin (Sigma-Aldrich), 30-nM sodium selenite (Sigma-Aldrich), and 20-nM progesterone (Sigma-Aldrich). Every 3 days, half of the medium was replaced with fresh N2 containing medium supplemented with 40-ng/mL hFGF-2 until cells reached confluence. The cells were normally passaged at a dilution of 1:5. HCNs were characterized by immunocytochemistry with nestin staining.

**Quantitative real-time PCR**

Quantitative real-time PCR was performed on a Bio-rad using the LightCycler 480 SYBR Green I Master Kit (Roche Diagnostics, Basel, Switzerland), according to the protocols recommended by the manufacturer. PCR cycling conditions were 94°C for 2 min (initial denaturation), then 45 cycles (94°C for 30 s, 60°C for 30 s, and 72°C for 30 s). The primer sequences for real-time PCR were: Ang II type-1 receptor (AT1R), sense 5'-GGAAACAGCTTGGTGGTGAT-3', antisense 5'-GTAAGATCGTCTTCTGCCAGC-3'; Ang II type-2 receptor (AT2R), sense 5'-GAAGGACAACTTCAGTTTTGC-3', and antisense 5'-GCATCTTATGTAGTTCCCCTTG-3'. Quantification was carried out by correcting for the amplification efficiency of the primer using a standard curve, followed by normalizing gene levels to the amount of the expressed β actin gene.

**Cell proliferation assay and viability test**

HCN cell proliferation was measured using the Cell Proliferation ELISA, 5-bromo-2-deoxyuridine (BrdU) colorimetric Kit (Roche Diagnostics). This assay quantifies the incorporation of BrdU, the thymidine analog, during DNA synthesis. HCN cells were seeded in 96-well plates at 5 × 10^3^ cells per well and cultured for 1–3 days. BrdU was added to each well to a final concentration of 10 µM for the last 4 h of culture. Cells were then fixed and DNA was denatured. Anti-BrdU-peroxidase conjugate was added and incubated for 90 min. After several washes, a color development ubstrate, tetramethylbenzidine, was applied and the absorbance was measured for 5 min at 370 nm using an enzyme-linked immunosorbent assay reader. In addition, cell death was determined by a Trypan blue exclusion assay (Gibco, Paisley, UK). Non-viable cells that were stained with Trypan blue (0.4%) and viable cells were counted with a hemocytometer. Each experiment was carried out in triplicate.

**Assessment of apoptosis using annexin V and propidium iodide** (**PI) flow cytometry**

The binding of annexin V–fluorescein isothiocyanate to externalized phosphatidylserine was used to measure apoptotic HCNs with an annexin V–PI apoptosis detection kit (BD Biosciences, NJ, USA) according to the manufacturer’s instructions. Samples were analyzed by flow cytometry within 1 h on a FACScan flow cytometer (BD Biosciences) and they were gated on the basis of forward versus side scatter for size, and the results are presented as the percentage of cells that were viable (Annexin V−) or nonviable (Annexin V+).

**Western blotting analysis**

HCNs were harvested at indicated time points and lysed in RIPA buffer [1% NP-40, 0.5% Na-deoxycholate, 0.1% SDS, 0.15-M NaCl, and 0.05-M Tris-HCl (pH8.0)] for 30 min on ice. After clearing by centrifugation at 12,000g for 10 min, the protein concentrations of the cell lysates were determined using a BCA kit (Thermo scientific, Waltham, MA, USA). Different samples with an equal amount of protein (10 µg) were separated on 10% SDS polyacrylamide gels, transferred to nitrocellulose membranes, and blocked in a blocking solution of 5% bovine serum albumin (BSA), 0.1% Tween 20, and TBS, at a pH of 7.4. Membranes were incubated overnight at 4°C with the primary antibodies. The primary antibodies used were as follows: Bcl-Xl (Cell Signaling Technology, Beverly, MA, USA); Bax (Cell Signaling Technology); AMP-activated protein kinase **(**AMPK) (Cell Signaling Technology); phospho-AMPK (Cell Signaling Technology); ACC (Cell Signaling Technology); phospho-ACC (Cell Signaling Technology); PGC1α (Santa Cruz Biotechnology, CA, USA); and β actin (Sigma-Aldrich). After rinsing with a blocking solution, the membranes were incubated with a horseradish peroxidase (HRP)-conjugated secondary antibody (1:3,000, Bethyl Laboratories, Montgomery, TX, USA) for 1 h at room temperature. After washing, protein bands were detected with a chemiluminescent HRP substrate (Thermo Scientific), as described by the manufacturer.

**Measurement of ROS**

The dichlorofluorescein diacetate (DCF-DA) method was used to measure intracellular ROS levels. The DCF-DA solution (25 μM, Invitrogen) was added to the HCNs and incubated for 10 min. The fluorescence of the 2', 7'-dichlorofluorescein product was determined using FACScan flow cytometry. To measure mitochondrial ROS, the mitochondria targeted, O_2_ sensitive, hydroethidine analog probe MitoSOX (Invitrogen) was used to determine relative O_2_ levels. Briefly, cells were harvested, washed in PBS, and resuspended in a final concentration of 1-μM MitoSOX to measure the mitochondrial superoxide (Invitrogen). Cells were incubated in MitoSOX for 30 min at 37°C in the dark. Stained cells were washed twice with PBS and then analyzed by FACScan flow cytometry.

**MitoTracker staining**

The mitochondrial cellular content was evaluated using MitoTracker Probes (Invitrogen) as follows. The cells were incubated for 10 min at 37°C in fresh culture medium containing a MitoTracker solution (100 nM). After staining, the cells were washed in fresh, pre-warmed culture media for 10 min to decrease cytosolic background signals derived from phenol red. Specific mitochondrial labeling was confirmed by confocal laser scanning microscopy.

**Electron microscopy**

The cells were washed twice in PBS and fixed in 2.5% glutaraldehyde in 0.1-M cacodylatebuffer (pH 7.2). Fixed cells were then detached by gentle scraping, pelleted, post-fixed in 1% osmium tetroxide in 0.1-M cacodylate buffer and processed for transmission electron microscopy. Thin sections (65 nm) were examined by electron microscopy (JEM-1200EX; JEOL USA Inc., Peabody, MA, USA).

**Mitochondrial complex I activity**

NADH oxidation to NAD+ by complex I was analyzed using an immunocapture complex I enzyme activity assay. The complex I (NADH-CoQ reductase) activity was measured inmitochondrial particles prepared by sonicating, under a nitrogen atmosphere, the mitochondria of 1 × 10^6^ HCNs dissolved in a lysis buffer [0.05-M KCl, 2-mM MgCl_2_, 0.25-M Sucrose, and 0.2-M Tris-HCl (pH 7.5)]. The assay mixture contained 250-mM Sucrose, 1-mM EDTA, 2-mM KCN, 0.3-μM Antimycin A, and 150-μM coenzyme Q1. The mitochondrial sample was added to the assay mixture, and the reaction was started by the addition of 0.2-mmol/L NADH. The reaction was measured by following the decrease in NADH absorbance 340 nm with a diode-array spectrophotometer. NADH activity was calculated using an extinction coefficient of 6.22 mmol/L^-1^×cm^-1^.

**Oxygen consumption rate (OCR) measurement**

A seahorse extracellular flux analyzer was used (Seahorse Biosciences, Massachusetts, MA, USA) to measure the rate of oxidative phosphorylation in HCNs. We seeded 1 × 10^5^ cells/well in 24-well Seahorse assay plates. OCR measurements were performed after the cells were equilibrated to running media (XF medium supplemented with 10-mM glucose or 1-mM pyruvate) for 1 h. Three baseline OCR measurements were performed, followed by injection of oligomycin (1 μM), which inhibits ATP synthesis by blocking the proton channel of the Fo portion of the ATP synthase. The uncoupler FCCP (0.5 μM) was used to determine the maximal respiratory rate, and rotenone (1 μM) and antimycin A (1 μM) were injected to determine the non-mitochondrial oxygen consumption. Experimental treatments were performed on 3–4 wells of each plate as technical replicates and each experiment had at least three biological replicates. OCR was normalized for the number of counted cells in each well.

**Small interfering RNA (siRNA) treatment**

siRNA duplexes targeting HCNs (5'-GAGAAUUCAUGGAGCAAUA-3' and 5'-GAAGAGCGCCGUGUGAUUU-3') were obtained from GE Healthcare. HCNs cultured in Opti-MEM medium (Gibco, GrandIsland, NY, USA) were transfected with siRNA using the Lipofectamine RNAiMAX Reagent (Invitrogen). Mock siRNA (si-CTL) was used as the experimental control. After 6 h of transfection, the transfection medium was replaced with normal medium. Cells transfected with si-PGC1α and si-CTL were used for 24 h after the medium change. The transcriptional effect of a mock siRNA treatment was compared with non-treated HCNs (WT).

**Transthoracic echocardiography**

Echocardiography was performed 4 weeks after left anterior descending artery ligation to confirm HF. The rats were anesthetized in a previously described manner and received mechanical ventilation. Images were acquired with a 12-MHz transducer connected to a Vivid 7 echocardiography machine (GE medical, Milwaukee, WI, USA). M-mode and 2-dimensional echocardiographic images at the papillary muscle level were acquired with a frame rate of ≥200/s. The left ventricular end-diastolic dimension (LVEDD), LV end-systolic dimension (LVESD), and LV ejection fraction (LVEF) were measured. All parameters were evaluated on an average of three consecutive beats.

**Measurement of serum Ang II**

The serum (500 μL) was serially obtained from HF rats at 6, 8, and 10 weeks after the model generation. The serum concentrations of Ang II were measured by using commercial ELISA kits **(**Enzo Life Sciences GmbH, Lörrach, Germany) according to the manufacturer’s instructions.

**Y-maze task**

The Y-maze test was made of black-colored acryl and positioned at equal angles. Rats (25 weeks old) were habituated in the Y maze recording room for 30 min. Rats were placed at the end of the arm and allowed to move freely through the maze and to enter as many arms as they chose during 10 and 12 min sessions. Arm entry sessions were recorded when the hind paws of the rat were completely placed in the arm. Consecutive entry into three arms in alternative order was defined as successive entries on overlapping triplet sets and the alternation percentage was calculated as the ratio of actual to possible alternations (defined as the total number of arm entries, minus 2), multiplied by 100.

**Immunohistochemical assay**

Rats were transcardially perfused with PBS followed by 4% paraformaldehyde (PFA) in PBS. After immersion fixed with 4% PFA in PBS for 4 h, the brains were cryoprotected in 30% sucrose-PBS and then frozen with an optimal cutting temperature (OCT) compound. Brain tissue blocks (stored at −80°C) were cryosectioned through the coronal plane at 30-μm thickness. The sections were stored at 4°C in the storing solution (30% glycerol, 30% ethylene glycol in PBS). For TUNEL staining, the cryosectioned brain slices were placed on a slide glass and treated with protease K (100 µg/mL) for 15 min after which they were retrieved at 60°C for 30 min and washed in 0.1% Triton X-100. Following retrieval, sections were permeabilized in 1% Triton X-100 for 10 min and then labeled with terminal transferase and nucleotide mix for 1 h at 37°C, as recommended by the manufacturer (In Situ Cell Death Detection Kit, Fluorescein, Roche), with 1-µg/mL PI (Sigma) for 5 min. For immunostaining assay of anti-BrdU and double staining of BrdU/subtype cell markers, four slices of every 5th brain slice of the dorsal hippocampus between bregma −2.56 and −3.76 mm were immunostained. Incubation with 2 N HCl at 37°C for 30 min was added to denature the chromatin after permeabilization in 0.5% Triton X-100 for 20 min. For immunostaining with NeuN, the cryosectioned brain slices were permeabilized in 0.5% Triton X-100 for 20 min and blocked in 15% normal serum with 3% BSA (bioWORLD, Dublin, OH, USA) and 0.1% Triton X-100 for 2 h in a free floating condition. The sections were incubated for 16 h at 4°C with antibodies against NeuN (1:1000; Covance, NJ, USA). Secondary antibodies conjugated with Alexa Fluor 488 (1:1,000, Invitrogen), Cy2 (1:500, Jackson) and Cy3 (1:500, Jackson) were used. Nuclei were counterstained with 1-µg/mL PI (Sigma) for 5 min. Immunostained sections were scanned with a confocal laser microscope (LSM510, Carl Zeiss, Oberkochen, Germany). Over four animals from each group were used for immunohistochemical analysis.

**Quantification**

In the immunohistochemical analysis, the number of immunostained cells against specific antibodies were counted in confocal images of >8 hippocampal coronal sections. For each staining analysis, every fifth cryosection of the dorsal hippocampal brain tissue region (AP: between bregma −4.5 and −4.3 mm) were taken to immunostain. The number of immunostained cells was presented as the average number of cells in the hippocampal field of one brain slice. In brain slice samples of 1 day and 4 weeks after BrdU injections, TUNEL- and NeuN-positive cells were presented as the number of cells per hippocampal field (hippo. 10–12 confocal microscopic fields) and BrdU-positive cells as the number of cells per granular cell layer (GCL) of the dentate gyrus, pyramidal layer, or whole hippocampal area (10–12 confocal microscopic fields). Four to eight animals per group were used for staining.

**Supplemental Figure Legends**

**Supplemental Figure 1. RT-PCR showed the constitutive expression of Ang II type-1 and -2 receptors in cultivated HCNs.**

**Supplemental Figure 2. Ang II augmented the production of total intracellular ROS through the Ang II type-1 receptor.**

ROS fluorescence ratios were 1.07, 1.39±0.17 (^*^*P*<0.05, compared with the no treatment group), and 1.32±0.06 (*P=*0.079) at 24, 48, and 72 h after Ang II treatment (1 µM), respectively (left figure). An increase in total intracellular ROS induced by Ang II (1 µM) for 48 h was abolished by Losartan and N-acetyl cysteine (NAC) pretreatment. Intracellular ROS ratios were 1.39±0.15 (^***^*P*<0.001, compared with the no treatment group), 1.10±0.11 (^**^*P*<0.01, compared with the Ang II treatment group), and 1.10±0.05 (^**^*P*<0.01, compared with the Ang II treatment group) for the Ang II treatment group, Losartan pretreatment group, and NAC pretreatment group, respectively (right figure).

**Supplemental Figure 3. NADPH oxidase inhibitor did not decrease HCN apoptotic death after Ang II treatment.**

(A) BrdU incorporations after Ang II treatment with a 1 h pretreatment of NADPH oxidase inhibitor were 85.4%±0.7% (^**^*P*<0.01, compared with the no Ang II treatment), 89.8%±2.0% (*P=*0.070, compared with 0 µM of NADPH oxidase inhibitor), 85.9%±1.1% (*P=*0.732), and 84.9%±0.7% (*P=*0.877) of the control for 0, 1, 5, and 20 µM, respectively.

(B) The MitoSOX fluorescence ratio after Ang II treatment (3.25±1.00 of the control) was not changed following pretreatment with the NADPH oxidase inhibitor group (Apocynin, 5 µM) (3.18±0.98).

**Supplemental Figure 4. Ang II decreased the HCN proliferation similar to pioglitazone, a known PGC1α activator.**

(A) Pioglitazone decreased HCN proliferation in a dose-dependent manner. A significant decrease was observed at pioglitazone concentrations of 10 µM. Ang II treatment also decreased HCN proliferation. BrdU incorporations 48 h after Ang II treatment were 89.9%±2.3% (*P=*0.208) and 60.8%±7.2% (^***^*P*<0.001) of the control for 5µM and 10 µM, respectively (left figure). BrdU incorporations 48 h after a pioglitazone (1 µM) or Ang II (1 µM) treatment were 75.3%±2.7% (^***^*P*<0.001) and 83.6%±2.8% (^**^*P*<0.01) of the control, respectively. BrdU incorporation after pioglitazone and Ang II co-stimulation was 69.3%±4.6% (^**^*P*<0.01, compared with the Ang II treatment group) of the control. Their proliferation effects of HCNs did not appear to be synergistic (right figure). (B) The expression of PGC1α and Bax increased after Ang II and pioglitazone treatment. However, Ang II and pioglitazone did not show a synergistic effect in increasing PGC1α expression.

**Supplemental Figure 5. Learning and memory ability seemed to improve at 10 weeks after administration of Losartan in a rat model of HF.**

The Y‑maze task showed that the rats faced a choice in selecting a pathway in the Y‑shaped track and that this was dependent on short term memory. The tendency for spontaneous alterations in arm entries in 10 and 12 min sessions was lower in the HF group compared with the control and HF+Lo groups. The number of arm entries was not substantially different across all groups, indicating that the alteration behavior was not caused by generally increased movement or any environmental change.
