# Supplementary figures and images for: Angiotensin II Causes Apoptosis of Adult Hippocampal Neural Stem Cells and Memory Impairment Through the Action on AMPK‐PGC1α Signaling in Heart Failure
Source: Stem Cells Transl Med. 2017 Feb 28;6(6):1491–503. doi: 10.1002/sctm.16-0382 (PMC5689768; doi:10.1002/sctm.16-0382)

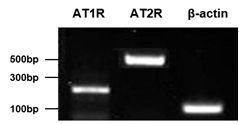

Supplement: Supplementary file 2 — Supporting Information [file SCT3-6-1491-s002.tif]

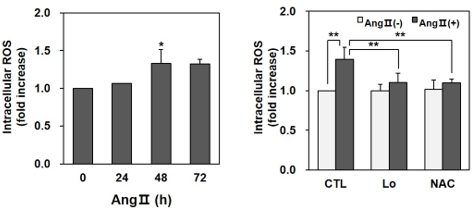

Supplement: Supplementary file 3 — Supporting Information [file SCT3-6-1491-s003.tif]

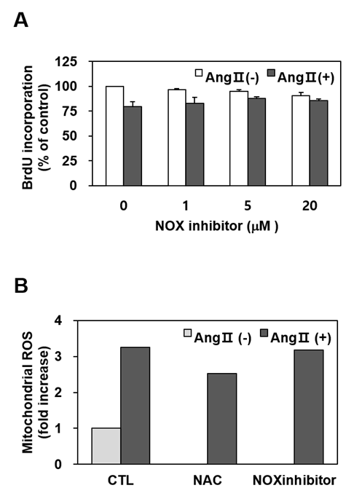

Supplement: Supplementary file 4 — Supporting Information [file SCT3-6-1491-s004.tif]

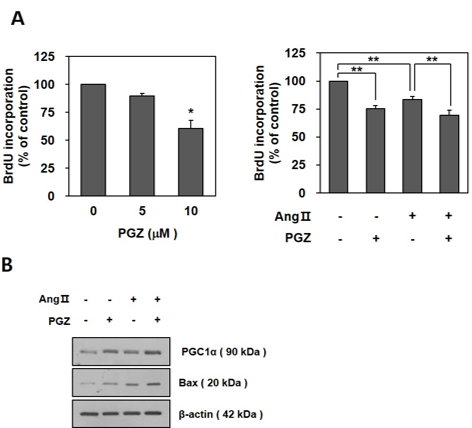

Supplement: Supplementary file 5 — Supporting Information [file SCT3-6-1491-s005.tif]

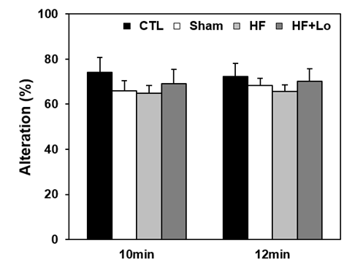

Supplement: Supplementary file 6 — Supporting Information [file SCT3-6-1491-s006.tif]
